# Glutathione-Loaded Solid Lipid Microparticles as Innovative Delivery System for Oral Antioxidant Therapy

**DOI:** 10.3390/pharmaceutics11080364

**Published:** 2019-07-27

**Authors:** Serena Bertoni, Beatrice Albertini, Carlotta Facchini, Cecilia Prata, Nadia Passerini

**Affiliations:** Department of Pharmacy and BioTechnology, University of Bologna, Via S. Donato 19/2, 40127 Bologna, Italy

**Keywords:** spray congealing, triglycerides, oral delivery, intestinal release, biorelevant media, antioxidant activity, reactive oxygen species

## Abstract

The present study aimed to develop a novel formulation containing glutathione (GSH) as an oral antioxidant therapy for the treatment of oxidative stress-related intestinal diseases. To this purpose, solid lipid microparticles (SLMs) with Dynasan 114 and a mixture of Dynasan 114 and Dynasan 118 were produced by spray congealing technology. The obtained SLMs had main particle sizes ranging from 250 to 355 µm, suitable for oral administration. GSH was efficiently loaded into the SLMs at 5% or 20% *w*/*w* and the encapsulation process did not modify its chemico-physical properties, as demonstrated by FT-IR, DSC and HSM analysis. Moreover, in vitro release studies using biorelevant media showed that Dynasan 114-based SLMs could efficiently release GSH in various intestinal fluids, while 2,2-diphenyl-1-picrylhydrazyl (DPPH) assay demonstrated the good radical scavenging activity of this formulation. Dynasan 114-based SLMs exhibited an excellent biocompatibility on intestinal HT-29 cells at concentrations up to 2000 μg/mL. SLMs containing GSH alone or together with another antioxidant agent (catalase) were effective in reducing intracellular reactive oxygen species (ROS) levels. Overall, this study indicated that spray congealed SLMs are a promising oral drug delivery system for the encapsulation of one or more biological antioxidant agents for local intestinal treatment.

## 1. Introduction

Oxidative stress is defined as an imbalance between oxidant and antioxidant species, with overproduction of reactive oxygen species (ROS) and other free radicals, a disruption of redox signaling and/or molecular damage [1]. The gastrointestinal (GI) tract is a key source of ROS production. Although the intestinal epithelium provides a tight chemical and physical barrier, ingested antigens and microbial pathogens can break through this protective layer, leading to inflammation and oxidative injury [2]. ROS overproduction has been implicated in the pathogenesis of diverse gastrointestinal diseases including inflammatory bowel disease (IBD), gastroesophageal reflux disease (GERD), gastritis, enteritis, colitis and associated cancers as well as pancreatitis and liver cirrhosis [3]. When the antioxidant capacity of the intestinal mucosa is compromised, the use of active substances able to decrease ROS can be beneficial, either associated or not with anti-inflammatory medicines [4].

**Figure 1:**
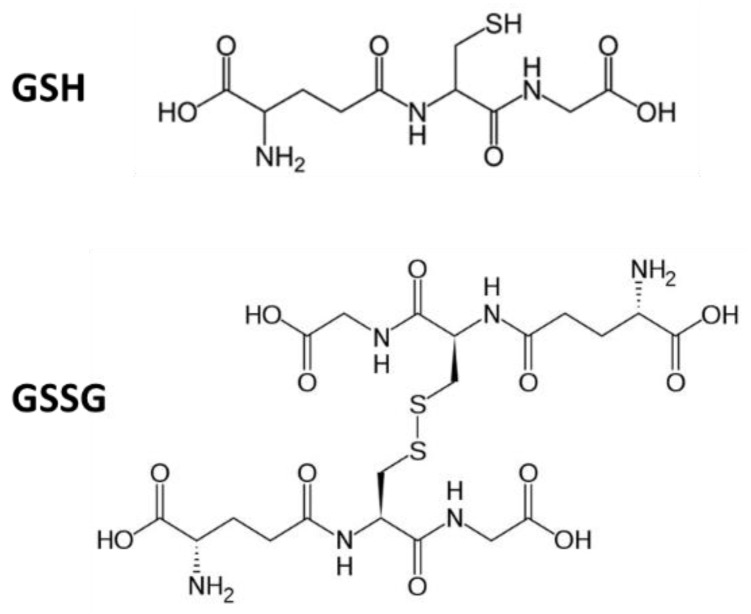
Chemical structures of glutathione (GSH) and glutathione disulfide (GSSG).

Glutathione (GSH) is a water-soluble tripeptide composed of the amino acids, glutamine, cysteine, and glycine (Figure 1). The thiol group is a potent reducing agent, making GSH one of the strongest physiological antioxidants [5]. Intracellular GSH exists mainly (98%) in the thiol-reduced form, while the oxidized form glutathione disulfide (GSSG, Figure 1) is usually around 1% [6]. However, in case of diseases and oxidative stress, the total GSH content and the GSH/GSSG ratio can drastically decrease [6]. Enhancement in GSH content can be a powerful way to prevent or treat a series of disorders associated with imbalance of the redox potential, ROS overproduction and oxidative stress. The supplementation of GSH by oral administration has proved to be effective in enhancing blood and GSH tissue levels in various animal models [7,8,9] and specifically to restore the intestinal mucosal GSH levels under conditions in which the antioxidant defenses are compromised [10]. Recent studies suggested that intact GSH can be rapidly uptaken by intestinal epithelial cells [11], and thus oral supplementation of GSH might be a powerful strategy to restore normal ROS levels in the intestine both in the extracellular and intracellular compartments at the diseased sites.

However, the oral administration of GSH is quite challenging, as the thiol group of GSH is highly reactive and can be oxidized both enzymatically and non-enzymatically to GSSG, which is devoid of antioxidant activity [12]. Moreover, as other peptides, GSH is likely to suffer from both chemical and enzymatic hydrolysis by digestive enzymes during gastrointestinal transit following oral administration, with the release of the free amino acids and loss of antioxidant activity. Thus, microencapsulation of GSH in a multiparticulate drug delivery system able to protect it from chemical and/or enzymatic degradation may enable the retention of its therapeutic properties following oral administration [13].

So far, the delivery systems explored for the GSH oral delivery have been limited to polymeric based-formulations. Trapani et al. developed Eudragit RS 100 microparticles loaded with GSH alone, together with a β-cyclodextrin [13] or other cyclodextrins [14] by an oil-in-oil (O/O) emulsion-solvent evaporation method. The same research group evaluated chitosan and chitosan/cyclodextrin nanoparticles as potential carrier systems for the oral delivery of this small peptide [15]. A plant-derived polymer (basil seed gum) was recently used to prepare nanoparticles loaded with GSH. These studies showed that with suitable formulation adjustments, the in vitro release of GSH could be modulated in order to limit its release in the stomach and enhance it in the intestine.

Lipid materials are nowadays receiving major attention due to their advantages in terms of biocompatibility and absence of toxicity, low-cost, suitability to be applied in solvent-free processes and ability to control the drug release. Specifically, previous studies showed that solid lipid microparticles (SLMs) based on Dynasan 114 (glyceryl trimyristate, C14) are promising vehicles for the encapsulation and oral delivery of proteins [16,17]. Moreover, SLMs can be produced by spray congealing, a solvent-free, fast, low-cost manufacturing technology [18]. Therefore, this study aims to encapsulate GSH in spray congealed triglycerides-based SLMs and to evaluate their potential as oral antioxidant therapy in the treatment of oxidative stress-related intestinal diseases. Specifically, a triglyceride with longer hydrophobic chains (Dynasan 118, glyceryl tristearate, C18) and less subject to lipolysis [16] than Dynasan 114 was added into the SLMs composition in order to achieve a prolonged GSH release in the intestinal tract, which is desired in case of inflammation. After loading of GSH into SLMs, their physicochemical characteristics (particle size and morphology, drug loading, solid state properties) and release profiles were evaluated. In these studies, biorelevant media were used instead of simple aqueous buffer solutions, which are typically used for dissolution testing but do not represent all the aspects of physiological conditions in the GI tract [19]. Finally, the best formulation was used to perform in vitro cytotoxicity studies on HT29 cell line, in order to assess the biocompatibility of SLMs, and its antioxidant activity was evaluated by measuring the intracellular ROS production of cells both in basal and oxidative stress conditions.

## 2. Materials and Methods

### 2.1. Materials

l-Glutathione reduced (GSH, powder ≥98.0%, m.w. 307.32 g/mol), l-Glutathione oxidized (GSSG, ≥98.0%), 1,4-Naphthoquinone (NPQ, 97%), 2,2-diphenyl-1-picrylhydrazyl (DPPH) and Catalase from bovine liver (CAT, lyophilized powder, 2000–5000 units/mg) were purchased from Sigma Aldrich (Steinheim, Germany). SLMs were prepared with Dynasan 114 or trimyristin (C14) and Dynasan 118 or tristearin (C18), which were obtained from Sasol (Witten, Germany). The colon cancer cell line HT29 was purchased from the American Type Culture Collection (Manassas, VA, USA). For cell culture, RPMI 1640 medium was obtained from Labtek Eurobio (Milan, Italy), fetal calf serum from Euroclone (Milan, Italy) and glutamine, methylthiazolyldiphenyl-tetrazolium bromide (MTT) and 2′,7′-Dichlorofluorescin diacetate (DCFH-DA) were purchesed from Sigma-Aldrich (St. Louis, MO, USA).

### 2.2. Preparation of Solid Lipid Microparticles (SLMs)

Two different MPs formulations were produced by spray congealing using the WPN atomizer [20]. In the first one (F1), Dynasan 114, was heated up to about 70 °C, while in the second formulation (F2) Dynasan 114 and Dynasan 118, at 1:1 weight ratio, were heated at about 75 °C. SLMs were prepared either without drug (unloaded-F1 and unloaded-F2, respectively), and with GSH at 5% *w*/*w* (F1 and F2, respectively). Additionally, a formulation based entirely on Dynasan 114 was loaded with 20% *w*/*w* (F3). GSH was added as powder into the melted carriers to form a suspension by gentle agitation. The nozzle temperature and the atomization pressure were set at 5 °C above the carrier melting point and at 1.5 bar, respectively. Upon loading the suspension into the feeding tank, the atomization allowed to the formation of droplets which solidified in the cooling chamber, kept at room temperature. The solid particles were collected and stored at 4 °C.

### 2.3. HPLC Analysis of GSH

The HPLC system consisted of two mobile phase delivery pumps (LC-10ADvp, Shimadzu, Japan), a UV–vis detector (SPD-10Avp, Shimadzu, Japan) and an autosampler (SIL-20A, Shimadzu, Japan).

Direct method: An HPLC method for the simultaneous quantification of GSH and GSSG was developed. A reversed phase C18 column (Synergi Hydro RP, 4 μm, 150 mm × 4.60 mm; Phenomenex, Bologna, Italy) was used as stationary phase, while the mobile phase consisted in 95% potassium phosphate buffer (25 mM, pH 2.7) and 5% methanol. The chromatographic run was carried out in isocratic mode with a mobile phase flow rate of 0.7 mL/min, while the injection volume and the detection wavelength were 20 µL and 200 nm, respectively. An example of chromatogram and calibration data are reported in Figure S1, Supplementary Materials. The limit of detection (LOD) and the limit of quantification (LOQ) are reported in Table S1, Supplementary Materials.

Derivatization method: For more complex media than simple saline buffers (e.g., biorelevant media), the direct method was not suitable for GSH determination because of the high signal of the matrix. Thus, a method based on the derivation of GSH by NPQ on the thiol group of GSH was carried out using a convalidated procedure [21]. Borate buffer solutions (pH 7.5; 250 mM) were prepared dissolving boric acid in water and adjusting the pH with sodium hydroxide. The derivatization reagent solution was prepared by dissolving NPQ (10 mM) in MeOH. An aliquot of 100 μL of the solution containing GSH (standard or sample) was added to 400 μL of borate buffer. To adjust the pH at 7.5. The derivatization reaction was started by the addition of 100 µL of NPQ solution and performed in a micro-reaction vial at room temperature. After 2 min, the derivation was complete, and 1.2 mL of mobile phase was added. The stationary phase was a C18 column (Kinetex, 5 μm, 150 mm × 4.60 mm; Phenomenex, Bologna, Italy), the mobile phase consisted in 60% potassium phosphate buffer (25 mM, pH 2.7) and 40% methanol with flow rate of 0.8 mL/min. The injected volume was 20 μL and the detection wavelength was set at 420 nm. An example of chromatogram and calibration data are reported in Figure S2, Supplementary Materials.

### 2.4. GSH-Loaded SLMs Characterization

#### 2.4.1. Drug Content Determination

20 mg of sample were accurately weighed and added to 5 mL of phosphate buffer pH 2.7. The system was heated to melt the carrier, shaken for 1 h and filtered to obtain a clear solution, which was assayed by HPLC using the direct method.

#### 2.4.2. Morphological Analysis

Shape and surface morphology of SLMs before and after dissolution testing were studied by scanning electron microscopy (SEM) using the microscope ESEM Quanta 200 (Fei Company—Oxford Instruments) at low accelerating voltage (20.0 kV). Double-sided adhesive black tape was used to fix the SLMs on the sample holder. The size distribution of the SLMs was evaluated by sieve analysis, using a vibrating shaker (Octagon Digital, Endecotts, London, UK) and four standard sieves (Scientific Instruments, Milan, Italy) of 100, 150, 250 and 500 μm. The more representative fraction was used for further experiments.

#### 2.4.3. Differential Scanning Calorimetry (DSC)

DSC analysis was carried out using a Perkin-Elmer DSC 6 (Perkin Elmer, Beaconsfield, UK). Before measurements, the instrument was calibrated with indium and lead for the temperature, and with indium for the enthalpy. Six to ten milligrams of samples were placed into aluminum pans and analyzed by DSC under a nitrogen flow of 20 mL/min. The analysis was carried out heating the samples from 25 to 250 °C at a scanning rate of 10 °C/min.

#### 2.4.4. Hot Stage Microscopy (HSM) Analysis

HSM studies were performed by means of a hot stage apparatus (Mettler-Toledo S.p.A., Novate Milanese, Italy) mounted on Nikon Eclipse E400 optical microscope. Images were taken using a Nikon Digital Net Camera DN100 connected to the microscope at a magnification of 10×. The samples were equilibrated at room temperature for 1 min and the physical changes were monitored during heating at a scanning rate of 10 °C/min.

#### 2.4.5. Stability of GSH in Gastric and Intestinal pH

GSH (5 mg) was added to 20 mL of buffer pH 1.2 and pH 6.8 and incubated under bland agitation at 37 °C. At determined time intervals, 1 mL of dissolution media was withdrawn, the sample was simply diluted and analyzed by HPLC using the direct method.

#### 2.4.6. In Vitro Release Studies of SLMs in Simulated Intestinal Fluids

Dissolution studies were performed both in compendial (buffer pH 6.8) and biorelevant media. Fasted State Simulated Intestinal Fluid (FaSSIF V2) and Fed State Simulated Intestinal Fluid (FeSSIF V2) were prepared according to Jantrid et al. [22]. About 60 mg of SLMs were immersed in 15 mL of the dissolution medium and incubated under bland agitation at 37 °C. At determined time intervals, 0.5 mL of dissolution media was withdrawn by means of a membrane filter (10 µm size) to not remove the SLMs. Fresh medium (0.5 mL) was added to keep the total volume constant. In case of simple pH 6.8 buffer, the sample was simply diluted and analyzed by HPLC using the direct method. In case of FaSSIF V2 and FeSSIF V2, the derivatization method was employed. The sample was treated as described in the previous paragraph and assayed by HPLC.

#### 2.4.7. Measurement of DPPH Radical Scavenging Activity

To investigate the radical scavenging properties of GSH-loaded SLMs, DPPH was dissolved in ethanol to a final working concentration of 0.2 mM. DPPH solutions (3.0 mL) were supplemented with 5 mg of SLMs (unloaded F1 and F2, GSH-loaded F1 and F2) in micro-vials, protected from light with aluminum foil and incubated at 37 °C for the 1 h. Additionally, free GSH was used as positive control, used at final concentration of 0.3 mM and 1.0 mM (corresponded to the GSH loaded into SLMs at 5% and 20%, respectively) either alone or added with unloaded SLMs. The samples were assayed for DPPH amount by measuring the absorbance at *λ* = 516 nm (UV2 Spectrometer, Unicam). Three repetitions were performed per sample. DPPH 0.2 mM served as 100% radical control, in comparison to the test samples. The ratio of free radical scavenging was calculated via normalization of the test sample absorbance to the absorbance of the DPPH alone as reported elsewhere [23].

### 2.5. Cell Culture

The human colon adenocarcinoma intestinal cell line (HT29), kindly provided by Prof. Natalia Calonghi (University of Bologna), were grown in RPMI 1640 medium supplemented with 10% fetal calf serum and 2 mM glutamine at 37 °C and 5% CO_2_. HT29 cells were seeded at 2 × 10^4^ cells/cm^2^ in a plastic well (60 cm^2^) and exposed to treatments after 1 day from the seeding.

### 2.6. MTT Assay

In order to estimate the effect of MPs on cell viability, MTT assay was performed as the reduction of tetrazolium salts to formazan is widely accepted as a reliable way to examine cell viability/proliferation [24]. Cells (2 × 10^4^/cm^2^) were incubated with different concentrations (50–2000 µg/mL) of unloaded-MPs (F1 formulation, fraction with diameters between 100 and 200 μm) for 24 h at 37 °C. Cells were then incubated with 5 mg/mL MTT for 4 h at 37 °C. Purple formazan salt crystals, formed during cell incubation, were dissolved by adding the solubilization solution (10% SDS, 0.01M HCl). Plates were incubated overnight in humidified atmosphere (37 °C, 5% CO_2_) and the absorbance was measured in a multi-well plate reader (Wallac Victor2, Perkin-Elmer) at 570 nm.

### 2.7. Measurement of Intracellular ROS Levels

To evaluate intracellular ROS levels, cells (1.5 × 10^4^/cm^2^) were incubated in the presence or absence of 100 µM H_2_O_2_ for 24 h and treated for 6 h with the SLMs (2000 µg/mL) in the dark, gently shaking every 20 min, in order to prevent sedimentation. Afterwards, cells were washed twice in HBSS and incubated with 5 µM DCFH-DA for 20 min at 37 °C. DCFH-DA, a small nonpolar and nonfluorescent molecule, is able to diffuse through the plasma membrane. Inside the cells, it is enzymatically deacetylated by intracellular esterases to a polar nonfluorescent compound, which is oxidized to the highly fluorescent 2,7-dichlorofluorescein (DCF). DCF fluorescence was detected using a multi-well plate reader (GENios, Tecan) at excitation and emission wavelengths of 485 nm and 535 nm, respectively. The samples used for intracellular ROS experiment are reported in Table S2, Supplementary Materials.

### 2.8. Statistical Analysis

All results were expressed as mean ± standard deviation (S.D.). One-way analysis of variance (ANOVA) followed by the Bonferroni post-hoc test (GraphPadPrism, GraphPad software Inc., CA, USA) were used to analyze the data and the level of significance was set at the probabilities of * *p* < 0.05, ** *p* < 0.01 and *** *p* < 0.001.

## 3. Results and Discussion

### 3.1. GSH-Loaded SLMs Characterization

SLMs with Dynasan 114 (F1) were prepared as reference formulation, based on previous results [16]. Additionally, a formulation with Dynasan 114 and Dynasan 118 in ratio 1:1 (F2) was prepared to evaluate the effect of a more hydrophobic excipient on the release characteristics of SLMs. The drug loading values (Table 1) were very close to the theoretical ones (5% *w*/*w*), thus demonstrating once again the high encapsulation efficiency of spray congealing process [18].

**Figure 2:**
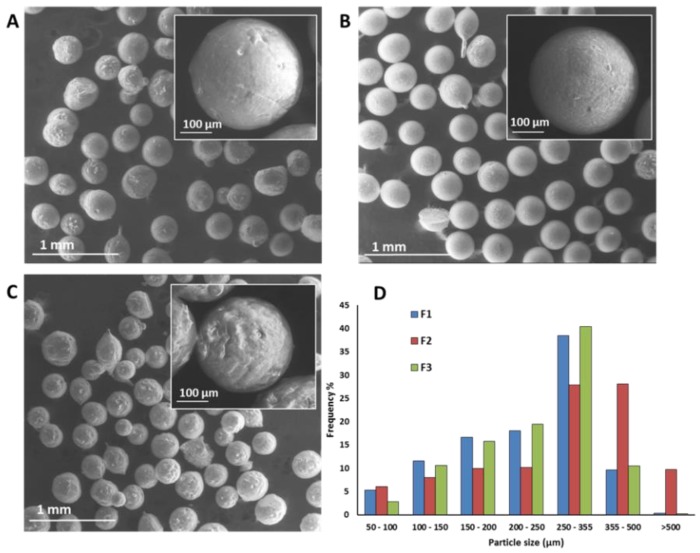
SEM images of GSH-loaded (**A**) F1, (**B**) F2 and (**C**) F3 SLMs. (**D**) Particle size distribution of GSH-loaded SLMs.

As visible in Figure 2A–C, SLMs were spherical and not aggregated. SLMs obtained by spray congealing technology are matrix systems in which the active substance is evenly dispersed into the carrier. Whereas F1 showed some irregularities, the F2 particle surface was more even and smooth due to the different lipid composition. F3 displayed a rougher surface due to the higher drug loading.

The particle size distribution of SLMs, reported in Figure 2D, showed Gaussian profile for all formulations. F1 and F3 SLMs had diameters comprised between 50 and 500 µm, with prevalent size fraction 250–355 µm. In case of F2 SLMs, the size distribution shifted to higher dimensions and the prevalent size fractions were the 250–355 µm and the 355–500 µm, as well. Therefore, for the subsequent characterization the 250–355 µm size fraction was selected.

**Figure 3:**
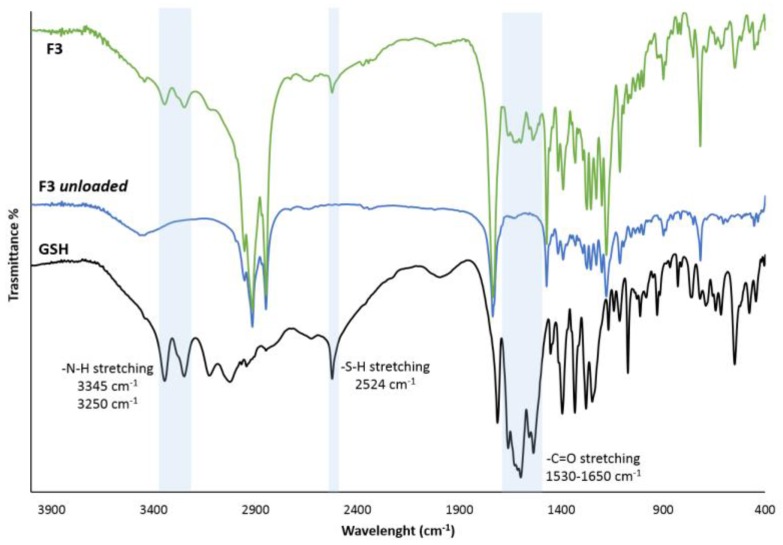
FT-IR spectra of GSH, F3 unloaded SLMs and F3 SLMs. The area of interest is evidenced in light blue.

Eventual peptide-carrier interactions or chemical modifications after the manufacturing process were then assessed by FT-IR analysis (Figure 3) on the formulation with higher GSH loading (F3) to clearly identify the GSH characteristic peaks. GSH gives a sharp band 2524 cm^−1^, which belongs to the characteristic S–H stretching vibration [25,26,27]. The two bands at 3345 cm^−1^ and 3250 cm^−1^ can be assigned to the N–H stretching [26]. The bands at about 1530–1650 cm^−1^ are due to the various C=O stretching vibrations and specifically the one at 1661 cm^−1^ belongs to the amide (amide I) group, whereas the strong band at 1713 cm^−1^ is attributed to the C=O stretching of the carboxyl group [25,27].

The FT-IR spectrum of F3 SLMs evidenced the presence of the band at 2524 cm^−1^, indicating that after incorporation of GSH into SLMs the thiol group of GSH maintained its integrity without being oxidized to S–S bond. Also, the other characteristics bands of GSH related to N–H and C=O stretching vibrations can be observed at the same wavenumbers. Additionally, the typical signals of the triglycerides of Dynasan 114 [28] can be detected both in loaded and unloaded SLMs.

**Figure 4:**
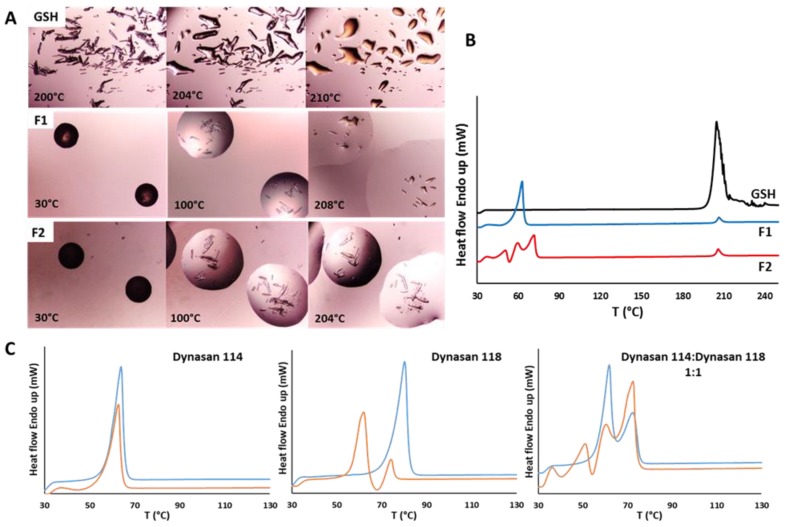
(**a**) Hot stage microscopy (HSM) images of GSH, F1 GSH and F2 GSH SLMs. The magnification was 10×. (**b**) Differential scanning calorimetry (DSC) analysis of GSH-loaded SLMs. (**c**) DSC of Dynasan 114, Dynasan 118 and Dynasan 114:Dynasan 118 (1:1 ratio) raw materials (blue curves) and SLMs prepared with the same excipients (orange curves). In case the of the Dynasan 114:Dynasan 118 (1:1 ratio), the sample was prepared as a physical mixture of the two excipients.

The HSM analysis of pure GSH (Figure 4a) showed needle-like crystals which melted at 203–205 °C, followed by a change to darker color, suggesting a probable decomposition. In case of SLMs (Figure 4a), the melting of the carrier was observed starting from 55 and 64 °C for F1 and F2, respectively. Afterwards, GSH crystals were observed into the melted particle matrix. Notably, the GSH crystals loaded into the SLMs showed unchanged morphology and melting temperature. It was interesting to notice that after melting, GSH was not miscible with the molten lipid, and the system remained as two liquid separate phases. In accordance with the HSM data, the DSC analysis (Figure 4b) of GSH showed a sharp endothermic peak at 205 °C due to the melting of the drug. The same event at the same temperature was detected in both formulations of SLMs, despite the limited amount of GSH (5%) in the samples. In addition, F1 SLMs showed a single endothermic peak at 63 °C attributed to the carrier melting, whereas in case of F3 the thermogram resulted much more complex, due to presence of two different components (Dynasan 114 and Dynasan 118). Moreover, additional DSC signals can originate from the formation of metastable polymorphic forms of the lipid excipient. In fact, long-chains triglycerides exist at least in three polymorphic forms identified by the lateral packing of their alkyl chains, the stable β form, and the metastable α and β’ forms [29]. As a matter of fact, melting-based processes can promote the conversion into the metastable forms. Four different endothermic signals related to the carrier melting were detected in F3 formulation immediately after preparation. The peak at highest temperature (69 °C) can be attributed to the melting of the original polymorph of Dynasan 118. The small peak at the lowest temperature (around 37 °C) corresponded to the melting of the metastable α form of Dynasan 114 [30]. In between, two more signals can be observed at 51 and 60 °C, respectively. To better understand the DSC results of F3 formulation, DSC analysis of single raw materials, physical mixture Dynasan 114:Dynasan 118 (ratio 1:1) and correspondent SLMs were performed (Figure 4c). The endothermic event at ca. 60 °C can be attributed both to Dynasan 114 original β form (Tm of 63 °C) and to the metastable α form of Dynasan 118, formed during spray congealing process (Tm of 62 °C). To the best of our knowledge, no data in the literature have reported information about the endothermic event detected at ca. 51 °C. In the DSC analysis of F2 SLMs performed after 1 month, the small peak at 37 °C of the α form of Dynasan 114 was no longer detectable, due to the complete conversion of the excipient into the stable form, whereas the other three peaks were unchanged.

These results suggest that the microencapsulation process did not cause any degradation and/or modification in GSH solid state and any interactions between drug and carrier.

### 3.2. In Vitro Release Studies

**Figure 5:**
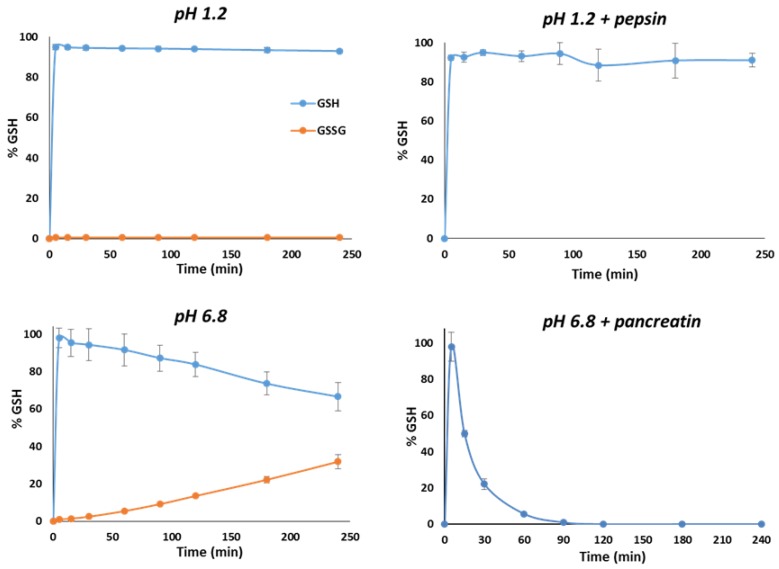
Stability profiles of GSH at pH 1.2, at pH 1.2 with addition of pepsin, at pH 6.8 and at pH 6.8 with addition of pancreatin. In case of simple buffers (pH 1.2 and pH 6.8) without enzymes, the amount of GSSG is also reported. Values are expressed as means (*n* = 3) ± SD.

To understand the behavior of GSH in the GI environments, dissolution studies of pure GSH were performed in buffer pH 1.2, simulating the gastric pH, and buffer 6.8, simulating the intestinal pH. Moreover, the effect of digestive enzymes on GSH stability was evaluated by adding pepsin and pancreatin to the gastric and intestinal fluids, respectively. As shown in Figure 5, in all conditions GSH completely solubilized within the first 5 min, confirming its high water solubility. However, whereas at acidic pH the reduced form was stable over 4 h, at pH 6.8 the amount of GSH decreased progressively due to its oxidation. In fact, greater amounts of GSSG were detected together with reduced GSH levels in simple buffer pH 6.8, while at pH 1.2 the percentage of GSSG was always lower than 1%. The pH-dependent oxidation of GSH is in accordance with previous studies [13,31,32].

Interestingly, whereas the addition of pepsin did not cause any substantial degradation of GSH, which remains around 90% during the 2 h incubation, a dramatic decrease in the GSH amount was observed at intestinal pH in the presence of pancreatic enzymes. The derivatization-based method employed for the quantification of GSH in the samples containing enzymes did not allow the quantification of GSSG, and thus it was not possible to follow the GSH oxidation in these conditions. However, we can hypothesize that the high instability of GSH in presence of intestinal fluid with pancreatin depends on a combination of GSH oxidation at neutral pH and the cleavage of the peptide bond by proteolytic enzymes. Another possible reason for GSH fast depletion is the formation of protein S-glutathionylation, caused by oxidation and/or disulfide exchange reactions at specific protein cysteinyl residues [33]. The formation of glutathione-adducts is related to the GSH function of the removal of radical, peroxides and many xenobiotic compounds [34].

**Figure 6:**
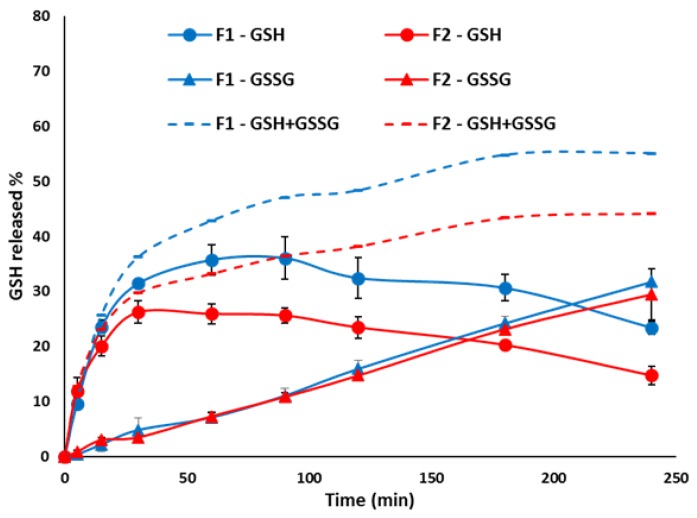
Release profiles of F1 and F2 SLMs in phosphate buffer pH 6.8. The experimental amount of GSH and GSSG was measured by HPLC (solid lines). The total amount of drug released, calculated by summing the amount of GSH and GSSG at every time points, is also reported (dotted line). Values are expressed as means (*n* = 3) ± SD.

The GSH released form SLMs with different composition (F1 and F2) was initially studied in buffer pH 6.8, simulating the intestinal pH (Figure 6). In the first 30 min, 31% and 26% of GSH was quickly released from F1 and F2, respectively, with a low amount of GSSG (<4%). This initial burst release, observed from both formulations, was followed by a plateau in the concentration of measured GSH, as the amount of GSH released form the particles was equivalent to the amount of GSH oxidized. In fact, an increasing amount of GSSG was detected. After 90 min, GSH concentration started to slowly decrease, indicating that the oxidation process was greater than the GSH release from the SLMs. Interestingly, the formulation composition did not seem to influence the amount of GSSG formed. However, the formulation F2 reduced the drug release, as expected, due to the more hydrophobic lipid carrier.

**Figure 7:**
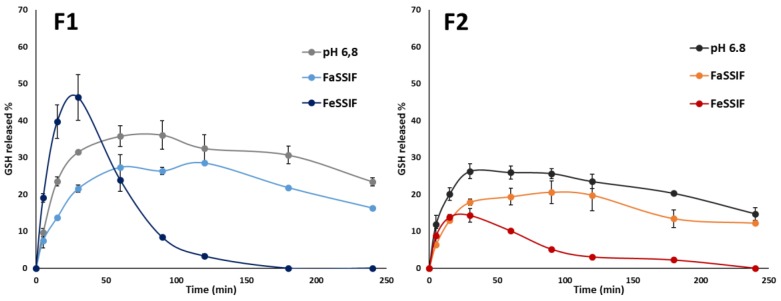
Release profiles of GSH from F1 and F2 SLMs in phosphate buffer pH 6.8, FaSSIF-V2 and FeSSIF-V2. Values are expressed as means (*n* = 3) ± SD.

After gastric transit, the small intestine is the first environment encountered by the microparticles. To improve our understanding of the GSH release, dissolution studies in biorelevant media simulating the small intestine were performed [35,36]. Figure 7 showed the amount of GSH released from F1 and F2 SLMs in FaSSIF and FeSSIF, compared to those released in simple buffer pH 6.8. In a fasted state, the GSH profile followed the same pattern of the one obtained in simple buffer, consisting of initial burst release, and a plateau phase followed by a slow decrease. However, compared to buffer pH 6.8, a slight reduction in GSH amount was observed in FaSSIF for both formulations, probably due to the higher viscosity of this medium compared to a simple aqueous buffer. In the case of F1 SLMs, the fastest drug release was observed in the medium simulating the fed state, in accordance with the release behaviour of a hydrophilic protein from Dynasan 114-based SLMs [16], where the highest drug released was observed in FeSSIF. This behaviour might be explained considering the effect of wettability and emulsification of lipid particles from the lecithin, monoglycerides and bile salts present in this medium. Around 50% of GSH was released in the first 30 min, followed by a fast GSH decrease, as was similarly observed for pure GSH in buffer pH 6.8 with pancreatin. Also, in this case, the fast GSH depletion can be favoured by the components of the medium, which can react with GSH to form a glutathione-adduct compounds. Differently, in case of formulation F2, the effect of enhanced GSH oxidation by FeSSIF prevailed over the enhanced release effect, and thus the total concentration of GSH measured during the test was quite low.

**Figure 8:**
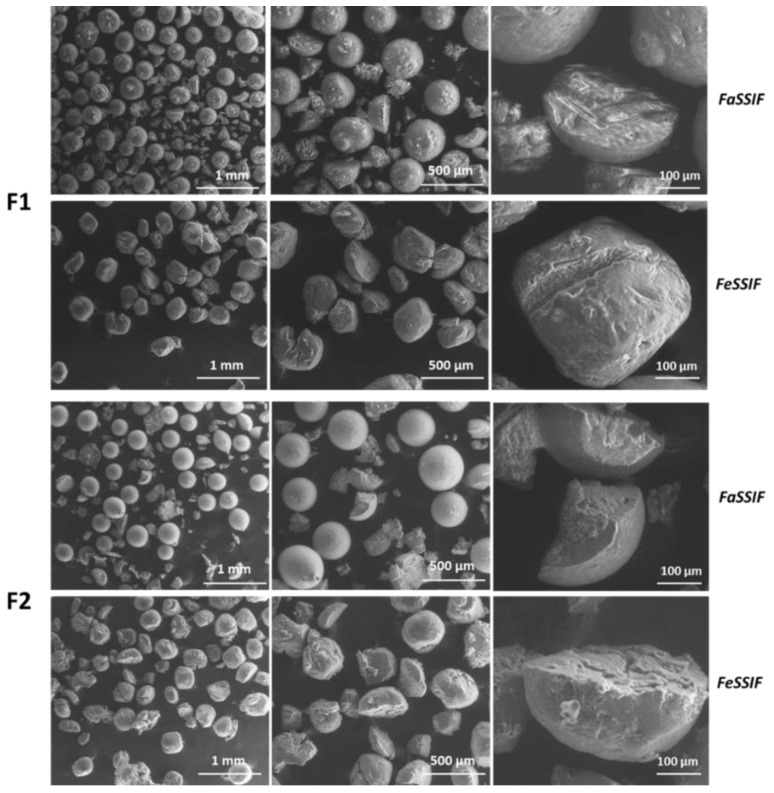
SEM images of the particles after the release test in FaSSIF-V2 and FeSSIF-V2, at three different magnifications (80×, 160× and 600×).

To gain more information about the dissolution behaviour of the SLMs, the morphology of the particles after release tests was observed by SEM and the images are reported in Figure 8. In the medium simulating the fasted state, the particles were partially intact and partially fragmented both in F1 and F2 SLMs. Differently, in the medium simulating the fed state, only a small amount of SLMs were recovered by the end of the test, indicating that most particles were completely emulsified within the medium. By observing the particles’ morphology, the loss of their spherical shape was evident, suggesting an erosion process of the lipid matrix resulting in a progressive degradation of the external surface of the SLMs. Moreover, numerous breakages and fractures were observed on the SLMs in this media, which were absent in the case of FaSSIF.

Therefore, the dissolution studies and SEM analysis performed at the end of the tests underlined important differences on the release of GSH and on the particle structure by using different biorelevant media. The critical stability of GSH in intestinal pH and its tendency to oxidize were evident, especially in presence of complex buffers. However, this does not necessarily mean that the antioxidant activity of GSH would be prevented. As mentioned, the thiol group of GSH is involved in reduction and conjugation reactions with mainly electrophilic or oxidizing species [37]. We should consider that in case of intestinal inflammation and other disease involving oxidative stress, the diseased sites are characterized by a high concentration of reactive species [38,39]. Therefore, upon GSH release close to the inflamed intestinal membrane, the released GSH would find a highly oxidizing environment and it would readily react with the excess of oxidant species. Clearly, it is important to verify the antioxidant activity of our formulations as well as their cytocompatibility with the intestinal cells.

### 3.3. Evaluation of Radical Scavenging Activity

**Figure 9:**
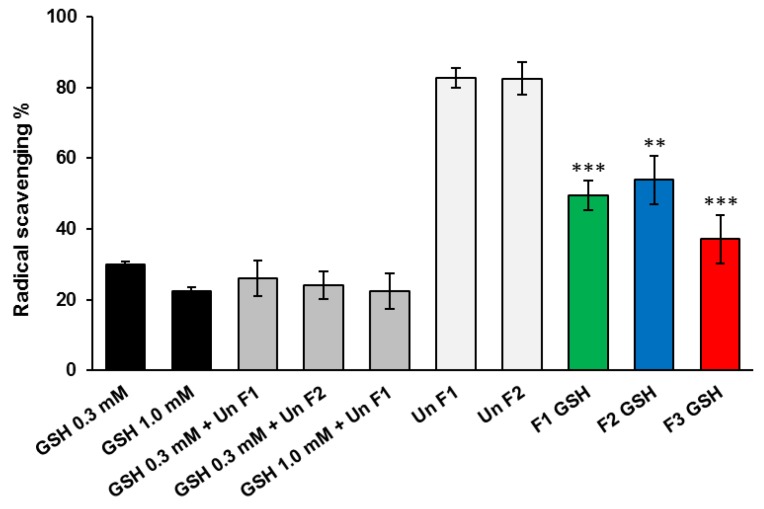
Antioxidant activity of free GSH (0.3 and 1.0 mM solutions), free GSH added with unloaded SLMs, unloaded SLMs and GSH-loaded SLM, measured with the decrease in absorbance of DPPH. Values are expressed as means (*n* = 3) ± SD, and the level of significance between GSH-loaded SLMs and corresponding unloaded SLMs was set at the probabilities of ** *p* < 0.01, and *** *p* < 0.001.

The ability of SLMs to scavenge ROS was studied using DPPH, which gives fast detection of the antioxidant activity towards radical species and the results are reported in Figure 9. The positive controls, consisting of GSH solutions at two concentrations (0.3 and 1.0 mM) corresponded to the GSH loaded into SLMs, and showed high radical scavenging activity against DPPH. As expected, the addition of unloaded SLMs to GSH solutions did not significantly affect the antioxidant potential of GSH (*p* > 0.05). Unloaded SLMs showed a slight effect on DPPH, with 82.7% and 82.4% of scavenging activity, for F1 and F2 SLMs, respectively. No significant differences between the two unloaded SLMs formulation were obtained. The antioxidant activity of GSH released from SLMs after 1 h of incubation was significantly stronger (*p* < 0.001 for F1 and F3 and *p* < 0.01 for F2) than that of unloaded formulations. Therefore, a good antioxidant activity towards radical species was observed, indicating the retention of anti-radical properties of GSH after microencapsulation. Specifically, the formulations with equivalent GSH content (5% *w*/*w*) resulted in a similar radical scavenging activity, equal to 49.4% and 53.9% for F1 and F2, respectively. F3, containing 20% *w*/*w* of GSH showed the highest antioxidant activity with a DPPH reduction of 37.1%.

### 3.4. Evaluation of the Effect of SLMs on Cell Viability

**Figure 10:**
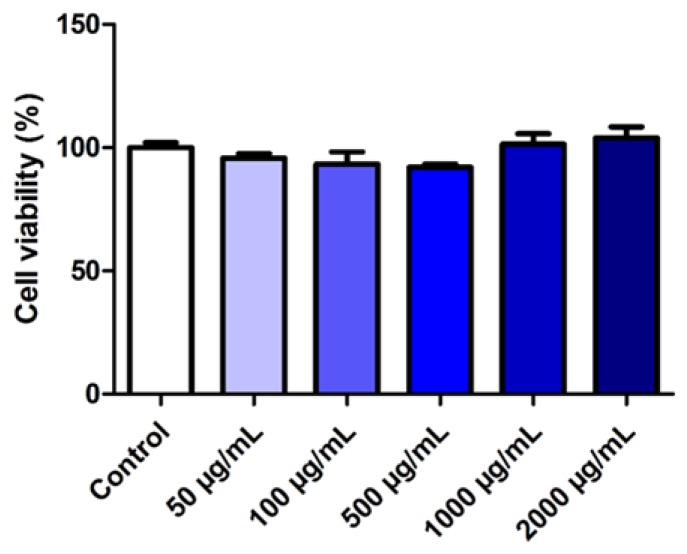
Viability of HT29 cells after 24 h incubation with different concentration of unloaded-F1 SLMs. Values are expressed as means (*n* = 4) ± SD.

To assess the biocompatibility of SLMs after oral administration, the cytotoxicity of the SLMs with the best performance (F3) was evaluated as drug-free (unloaded) formulation on human colon adenocarcinoma intestinal cell line (HT29). The HT29 cells represent a well-characterized model to study the intestinal epithelial response to drugs, food compounds, microorganisms as well as nano- or micro-delivery systems and they represent a valuable model due to their similarities with enterocytes of the small intestine [40]. As shown in Figure 10, concentrations ranging from 50 to 2000 μg/mL were completely safe on cells, with viability values always close to 100% (*p* < 0.05 compared to the control), demonstrating the excellent biocompatibility of SLMs.

### 3.5. Effect of GSH-Loaded SLMs on Intracellular ROS Levels

**Figure 11:**
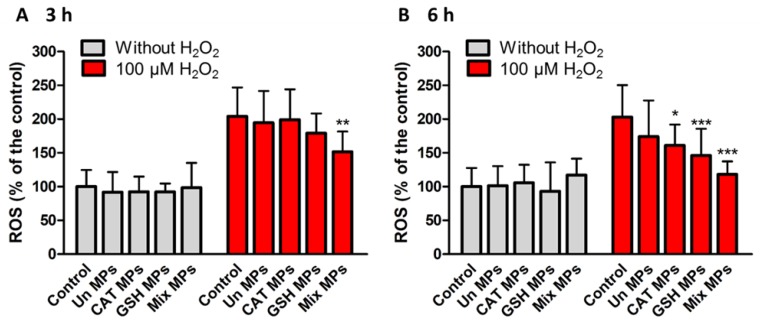
Intracellular ROS levels of HT-29 cells using DCFH-DA assay measured after (**A**) 3 h and (**B**) 6 h of incubation with cell medium (control), unloaded MPs (Un MPs) and the SLMs containing antioxidant agents (GSH MPs, CAT MPs and Mix MPs). Values are expressed as means (*n* = 6) ± SD and the level of significance was set at the probabilities of * *p* < 0.05, ** *p* < 0.01 and *** *p* < 0.001 compared to the control sample with the same pre-treatment.

Formulation F3, which showed the highest radical scavenging activity, was used to test the potential antioxidant activity on HT-29 cells. To simulate the basal and oxidative stress conditions of the intestine, cells were used without and with pre-treatment with 100 µm H_2_O_2_, respectively. In order to evaluate the potential of SLMs as vehicle for antioxidant therapy in ROS-related intestinal diseases, GSH-loaded SLMs F3 were compared with the same particles loaded with another antioxidant compound, catalase (CAT), which catalyzes the decomposition of hydrogen peroxide to water and oxygen. In fact, due to its H_2_O_2_ decomposition capacity, CAT has been proposed as an oral antioxidant therapy for various intestinal diseases [41]. In analogy to F3, CAT was encapsulated at 20% *w*/*w* in Dynasan 114-based SLMs. Additionally, a possible synergistic effect of GSH and CAT was evaluated by loading both actives into Dynasan 114-based SLMs at a percentage of 10% *w*/*w* of each agent, to maintain the total drug loading at 20% *w*/*w* (Table S2, Supplementary Materials). Figure 10 shows intracellular ROS levels measured after cell incubation with or without unloaded MPs (Un MPs) and the three SLMs containing the antioxidant agents either singularly (GSH MPs, CAT MPs) or in combination (Mix MPs) for 3 h (Figure 11A) and 6 h (Figure 11B). No significant difference in intracellular ROS was observed in the cells without H_2_O_2_ pre-treatment, indicating that the SLMs were ineffective in reducing ROS amount in basal conditions characterized by a physiological redox state. After H_2_O_2_ treatment, the ROS production increased more than two-fold (222% and 215% in the control samples measured after 3 and 6 h, respectively). Whereas Un MPs did not influence ROS amount, the SLMs containing antioxidant agents caused a significant decrease in ROS intracellular levels. Specifically, the antioxidant effects of CAT MPs and GSH MPs were detectable after 6 h (*p* < 0.05 and *p* < 0.001, respectively), while the formulation containing a combination of GSH and CAT was the most effective, showing a strong antioxidant activity already after 3 h (*p* < 0.01) and further reducing the ROS amount after cell incubation for 6 h (*p* < 0.01). The superior antioxidant power exerted by mix MPs compared to CAT MPs and GSH MPs could be attributed to a synergistic effect between GSH, with strong but unspecific radical scavenging activity, and CAT, whose enzymatic activity is specific and restricted to hydrogen peroxide. Moreover, various studies indicated that GSH is capable of entering the intestinal cells as an intact molecule [11] and thus we can hypothesize a direct antioxidant effect against intracellular radical species, resulting in a ROS scavenging power of a greater extent compared to CAT alone (Figure 11B). Differently, CAT is a hydrophilic macromolecule which is unlikely to be able to cross cell membrane and its effect would be mainly observed on extracellular ROS giving a less pronounced effect. However, the reduction of extracellular ROS can reflect on the intracellular ones, since H_2_O_2_ and other ROS can cross cellular membranes [42,43]. At the end of the 6 h incubation with the GSH-loaded SLMs, the viability of cells treated with H_2_O_2_ was restored (Figure S3, Supplementary Materials).

## 4. Conclusions

In this study reduced glutathione (GSH) was successfully encapsulated into SLMs by spray congealing. Dynasan 114 was used as main lipid carrier, alone (formulation F1) or in combination with a triglyceride with longer chains length, Dynasan 118 (formulation F2). SLMs showed excellent encapsulation efficiency values and no modification, loss or degradation of the tripeptide during the process. FT-IR, DSC and HSM analysis showed that GSH chemico-physical properties were maintained. Moreover, the variations in the lipid composition have the potential to modulate the drug release. Specifically, the presence of more hydrophobic lipid prolonged the drug release, whereas the fastest GSH release was obtained by F1 SLMs and in fed state simulated intestinal medium (FeSSIF). GSH-loaded SLMs showed radical scavenging activity, with the best performance obtained by GSH-loaded F1 SLMs. This formulation showed excellent biocompatibility on HT-29 cells at concentrations up to 2000 μg/mL. Finally, SLMs containing GSH alone or together with other antioxidant agents (catalase) were effective in reducing intracellular ROS in the presence of oxidative stress mimicked by hydrogen peroxide. Overall, spray congealed SLMs are confirmed to be a promising vehicle for the encapsulation and intestinal delivery of biotherapeutics.

## Figures and Tables

**Table 1:** Composition and GSH content of F1, F2 and F3 solid lipid microparticles (SLMs).

SLMs	Constituents (%, *w*/*w*)			Drug Loading (%, *w*/*w*)
	Dynasan 114	Dynasan 118	API	GSH
F1	95.0	-	5.0	5.09 ± 0.24
F2	48.5	48.5		5.18 ± 0.12
F3	80.0	-	20.0	21.66 ± 0.29

## Supplementary Materials

The following are available online at https://www.mdpi.com/1999-4923/11/8/364/s1, Figure S1: HPLC chromatograms of a standard GSH (Rt = 4.1 min) and GSSG (Rt = 6.3 min) solution (A); HPLC linearity: calibration plot, slope, y-intercept and determination coefficient of GSH (B) and GSSG (C); Figure S2: HPLC chromatograms of a standard GSH solution (Rt = 6.4 min), while the unreacted 1,4-Naphthoquinone (NPQ) is detected at Rt = 12.1 min (A); HPLC linearity: calibration plot, slope, y-intercept and determination coefficient of GSH (B); Figure S3: Viability of HT29 cells after incubation with SLMs for 6 h assessed by MTT assay (in the same experimental conditions used for the determination of intracellular ROS levels). Values are expressed as means (*n* = 4) ± SD. The level of significance between the Control sample treated with H_2_O_2_ and the Control without pre-treatment was set at the probability of ### *p* < 0.001. The level of significance between the GSH MPs and Mix MPs and the corresponding Control was set at the probabilities of **p* < 0.05, and ****p* < 0.001; Table S1: LOD and LOQ of GSH and GSSG by direct HPLC method; Table S2: Composition of SLMs used for intracellular ROS studies.
