# Supplementary material for: Glutathione-Loaded Solid Lipid Microparticles as Innovative Delivery System for Oral Antioxidant Therapy
Source: Pharmaceutics. 2019 Jul 27;11(8):364. doi: 10.3390/pharmaceutics11080364 (PMC6723327; doi:10.3390/pharmaceutics11080364)
Supplement: Supplementary file 1 [file pharmaceutics-11-00364-s001.pdf]

pharmaceutics

MDPI

Supplementary Materials: Glutathione-Loaded Solid Lipid Microparticles as Innovative Delivery System for Oral Antioxidant Therapy

Serena Bertoni, Beatrice Albertini, Carlotta Facchini, Cecilia Prata and Nadia Passerini

1. HPLC Analysis of GSH

Direct method. An example of chromatogram and the calibration plots are reported in Figure S1. The limit of detection (LOD) and the limit of quantification (LOQ) were determined with the signal-to-noise ratio method [1] and are reported in the Table S1.

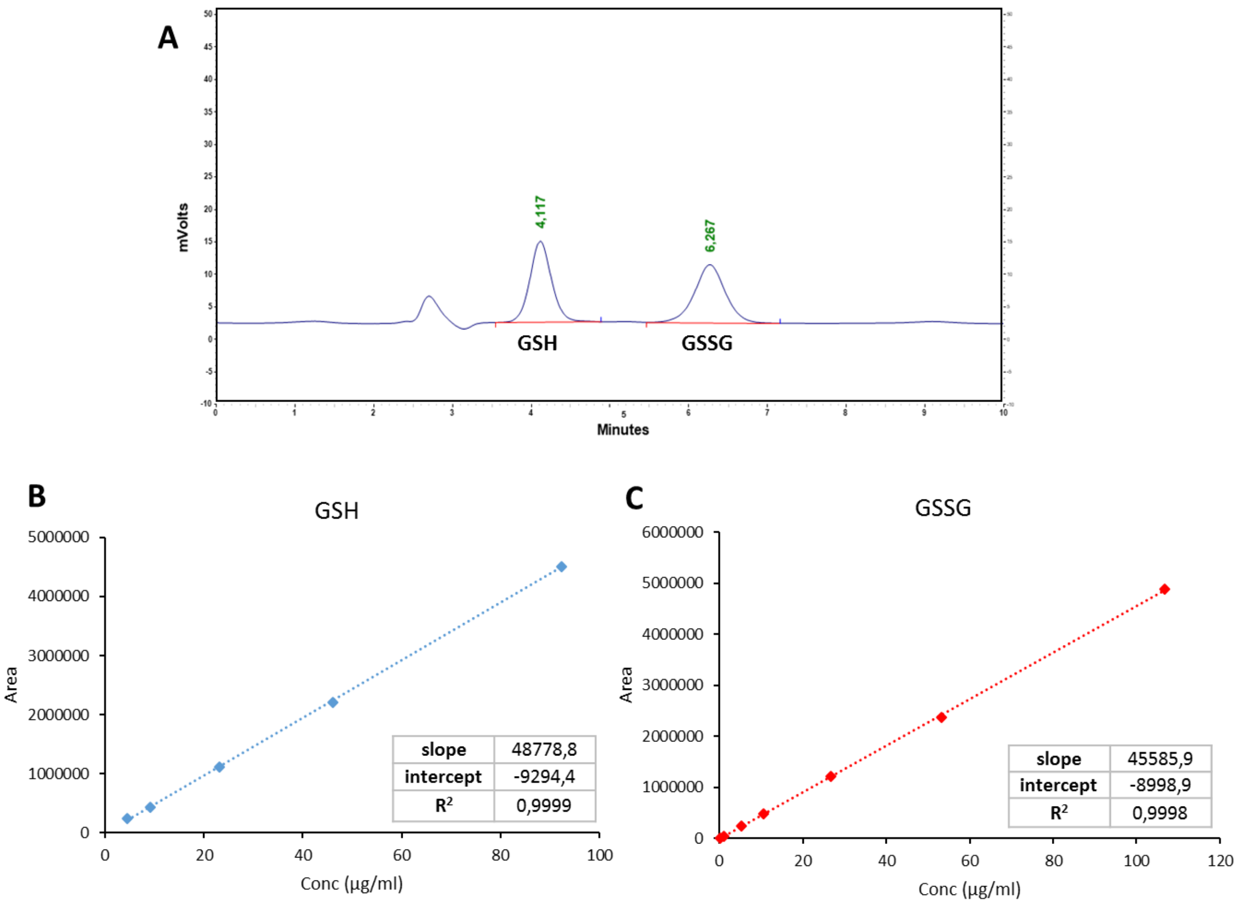

Figure S1. HPLC chromatograms of a standard GSH (Rt = 4.1 min) and GSSG (Rt = 6.3 min) solution (**A**); HPLC linearity: calibration plot, slope, y-intercept and determination coefficient of GSH (**B**) and GSSG (**C**).

Table S1. LOD and LOQ of GSH and GSSG by direct HPLC method.

	LOD (µg/ml)	LOQ (µg/ml)
**GSH**	0.028	0.092
**GSSG**	0.046	0.153

Derivatization method. An example of chromatogram and the calibration plot are reported in Figure S2.

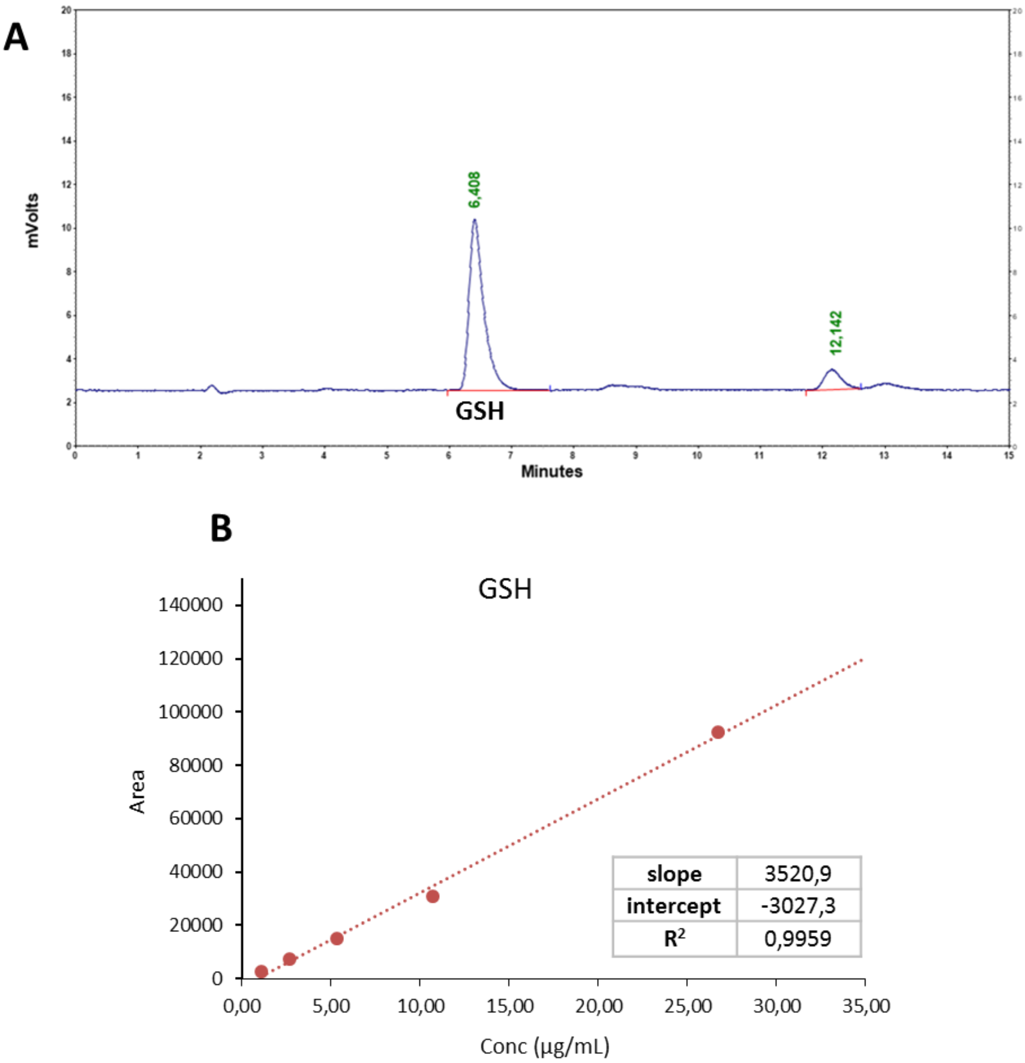

Figure S2: HPLC chromatograms of a standard GSH solution (Rt = 6.4 min), while the unreacted 1,4-Naphthoquinone (NPQ) is detected at Rt = 12.1 min (**A**); HPLC linearity: calibration plot, slope, y-intercept and determination coefficient of GSH (**B**)

2. Preparation of Solid Lipid Microparticles (SLMs) for Intracellular ROS Experiment

The antioxidant effect of 20% GSH-loaded SLMs was compared with SLMs containing another antioxidant compound, catalase (CAT) or a mixture of both compounds to have the same antioxidant concentration. The samples used for intracellular ROS experiment are schematized in Table S2.

Table S2. Composition of SLMs used for intracellular ROS studies.

SLMs	Constituents (%, *w/w*)		
	Dynasan 114	GSH	CAT
Un MPs	100.0	-	-
GSH MPs		20.0	
CAT MPs	80.0	-	20.0
Mix MPs		10.0	10.0

SLMs containing CAT and the mixture of both antioxidant compounds were produced using the same conditions of the spray congealing equipment employed for GSH MPs (temperature of the nozzle was 5 °C above the melting point of the carrier, and inlet air pressure was 1.5 bar).

Specifically, Dynasan 114 was melted at about 70°C. CAT or the mixture of GSH and CAT were added as powders and suspended into the melted carriers, prior to atomization.

3. Cell Viability after Treatment with SLMs in the Presence or Absence of Oxidative Stress Mimicked by H_2O_2

At the end of the 6 h incubation with the SLMs, the cell viability was measured and the results are reported in Figure S3. As expected, cell viability was always close to 100% for the cells in basal conditions, but decreased to about 80% in oxidative stress conditions ($p < 0.001$). Interestingly, the treatment with SLMs containing GSH ($p < 0.05$) and the combination of GSH and CAT ($p < 0.001$) was able to limit the cell damage by ROS overproduction and restore the cell viability, according to data obtained by the evaluation of intracellular ROS levels.

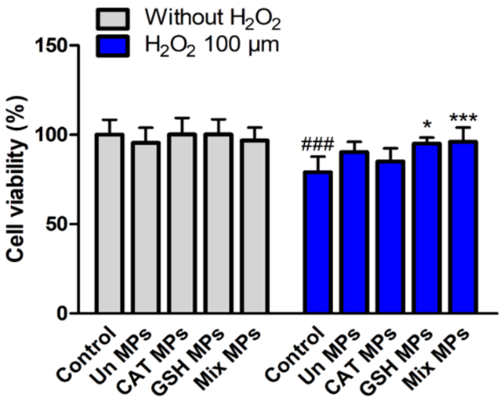

Figure S3: Viability of HT29 cells after incubation with SLMs for 6 h assessed by MTT assay (in the same experimental conditions used for the determination of intracellular ROS levels). Values are expressed as means ($n = 4$) ± SD. The level of significance between the Control sample treated with H_2O_2 and the Control without pre-treatment was set at the probability of $^{###}p < 0.001$. The level of significance between the GSH MPs and Mix MPs and the corresponding Control was set at the probabilities of $^{*}p < 0.05$, and $^{***}p < 0.001$.

References

[1] A. Shrivastava, V. Gupta, R. Article, Methods for the determination of limit of detection and limit of quantitation of the analytical methods, Chronicles Young Sci. 2 (2011) 21–25. doi:10.4103/2229-5186.79345.
